# The candidate genes *TAF5L, TCF7, PDCD1*, *IL6 *and *ICAM1 *cannot be excluded from having effects in type 1 diabetes

**DOI:** 10.1186/1471-2350-8-71

**Published:** 2007-11-28

**Authors:** Jason D Cooper, Deborah J Smyth, Rebecca Bailey, Felicity Payne, Kate Downes, Lisa M Godfrey, Jennifer Masters, Lauren R Zeitels, Adrian Vella, Neil M Walker, John A Todd

**Affiliations:** 1 Juvenile Diabetes Research Foundation/Wellcome Trust Diabetes and Inflammation Laboratory, Department of Medical Genetics, Cambridge Institute for Medical Research, University of Cambridge, Addenbrooke's Hospital, Hills Road, Cambridge, CB2 OXY, UK; 2 Metabolic Disease Group, Sulston Laboratory, Wellcome Trust Sanger Institute, Hinxton, Cambridge, CB10 1SA, UK; 3 Division of Transfusion Medicine, Department of Haematology, University of Cambridge, East Anglia Blood Centre, Cambridge, CB2 2PT, UK; 4 Mayo Clinic College of Medicine, Division of Endocrinology and Metabolism, 200 First ST SW, Rochester, MN55905, USA

## Abstract

**Background:**

As genes associated with immune-mediated diseases have an increased prior probability of being associated with other immune-mediated diseases, we tested three such genes, *IL23R*, *IRF5 *and *CD40*, for an association with type 1 diabetes. In addition, we tested seven genes, *TAF5L*, *PDCD1, TCF7, IL12B*, *IL6*, *ICAM1 *and *TBX21*, with published marginal or inconsistent evidence of an association with type 1 diabetes.

**Methods:**

We genotyped reported polymorphisms of the ten genes, nonsynonymous SNPs (nsSNPs) and, for the *IL12B *and *IL6 *regions, tag SNPs in up to 7,888 case, 8,858 control and 3,142 parent-child trio samples. In addition, we analysed data from the Wellcome Trust Case Control Consortium genome-wide association study to determine whether there was any further evidence of an association in each gene region.

**Results:**

We found some evidence of associations between type 1 diabetes and *TAF5L*, *PDCD1*, *TCF7 *and *IL6 *(ORs = 1.05 – 1.13; *P *= 0.0291 – 4.16 × 10^-4^). No evidence of an association was obtained for *IL12B*, *IRF5*, *IL23R, ICAM1, TBX21 *and *CD40*, although there was some evidence of an association (OR = 1.10; *P *= 0.0257) from the genome-wide association study for the *ICAM1 *region.

**Conclusion:**

We failed to exclude the possibility of some effect in type 1 diabetes for *TAF5L*, *PDCD1*, *TCF7*, *IL6 *and *ICAM1*. Additional studies, of these and other candidate genes, employing much larger sample sizes and analysis of additional polymorphisms in each gene and its flanking region will be required to ascertain their contributions to type 1 diabetes susceptibility.

## Background

Type 1 diabetes is a chronic autoimmune disease with a complex pathogenesis involving multiple genetic and environmental factors. Before the advent of genome-wide association (GWA) studies, disease loci were primarily sought through the testing of candidate genes, selected based usually upon limited prior information about the function of the gene and the pathogenic mechanisms of disease. The candidate gene approach has been successful in finding disease loci, but as only relatively small numbers of genes have been studied, few true positive associations have been found, despite numerous studies and enormous effort [1]. Only five type 1 diabetes loci with compelling evidence had been identified before the advent of GWA studies: the HLA class II genes on chromosome 6p21 [2]; the insulin gene (*INS*) on 11p15 [3]; *CTLA4 *on 2q33 [4]; *PTPN22 *on 1q13 [5,6]; and, *IL2RA/CD25 *on 10p15 [7,8]. Another five type 1 diabetes loci with convincing evidence have so far been identified by GWA studies in chromosome regions 2q24.3 [9], 12q24, 12q13, 16p13 and 18p11 [10,11].

Previously, we noted that, with the exception of *INS *[12], the type 1 diabetes loci contain polymorphisms that have been associated with susceptibility to other immune-mediated diseases, such as Graves' disease (GD) and systemic lupus erythemastosus (SLE), suggesting the existence of shared disease loci [13]. In this study, we tested three genes, namely, *IL23R*, *IRF5 *and *CD40*, which have been associated with other immune-mediated diseases (Table 1), including Crohn's disease (CD), psoriasis, SLE and GD, for an association with type 1 diabetes. In addition, we tested seven genes, namely, *ICAM1*, *TAF5L*, *PDCD1, TCF7*, *IL12B*, *IL6 *and *TBX21*, with marginal or inconsistent evidence of an association with type 1 diabetes (Table 1). *PDCD1 *has also been associated with SLE and Rheumatoid Arthritis (RA). We genotyped reported polymorphisms, nonsynonymous SNPs (nsSNPs) and tag SNPs for the *IL12B *and *IL6 *regions in up to 7,888 case, 8,858 control and 3,142 parent-child trio samples. In addition, we used Wellcome Trust Case Control Consortium (WTCCC) [10] GWA study data to determine whether there was any further evidence of an association with type 1 diabetes in the linkage disequilibrium (LD) blocks containing the reported polymorphisms of interest.

**Table 1:** Candidate genes and their previous associations.

Gene and polymorphism	Candidacy	Previous disease associations
*TAF5L *on 1q42 C744A (rs3753886)	*TAF5L *encodes a protein that is a component of the PCAF histone acetylase complex. It may participate in basal transcription, serve as a coactivator, function in promoter recognition or modify general transcription factors to facilitate complex assembly and transcription initiation.	T1D: ▪ Chistiakov *et al *[35] 247 Russian cases and 258 controls, derived OR for C allele = 0.69 (0.52–0.92), *P *= 0.013.

*PDCD1 *on 2q37 7146G>A (rs11568821) 872C>T (rs2227981)	Belongs to the B7-CD28 superfamily. These molecules play a critical role in the development of the immune response by controlling T cell numbers, through a fine balance of stimulation and negative regulation, which is essential for the prevention of autoimmunity.	SLE, RA and T1D: ▪ Prokunina *et al *[58] SLE: 7146G>A: 443 families and 520 cases of European, Mexican and African-American decent, RR for A allele = 2.6 (1.6–4.4), *P *= 1.0 × 10^-5 ^for Europeans and RR for A allele = 3.5 (1.4–8.5), *P *= 9.0 × 10^-4 ^for Mexicans. ▪ Prokunina *et al *[37] RA: 7146G>A: 1,175 cases and 3,404 controls, OR for A allele = 1.18 (0.99–1.41), *P *= 0.05. ▪ Lin *et al *[59] 872C>T: 98 SLE cases and 100 controls, 84 RA cases and 135 controls, OR for T allele = 3.32 (1.78–6.21), *P *< 0.0001, no association in SLE. ▪ Nielsen *et al *[36] T1D:7146G>A: 192 Danish cases and 155 controls, OR for A allele = 1.92 (1.1–3.3), *P *= 0.02.

*TCF7 *on 5q31 Pro19Thr (rs5742913)	Role as a transcription factor in regulating key immune response elements and its involvement in T-cell development in the thymus.	T1D: ▪ Noble *et al *[38] 282 USA families, RR = 1.21, *P*_TDT _= 0.12. A allele transmitted from fathers, RR = 1.79, *P*_TDT _= 0.007, *P*_ESP _= 0.03. To male offspring, *P*_ESP _= 0.04. To low HLA risk (non DR3/DR4) offspring, RR = 1.35, *P*_TDT _= 0.06, *P*_ESP _= 0.04. Early onset offspring, *P*_ESP _= 0.09.

*IL12B *on 5q33.3 A1159C (rs3212227) Microsatellite D5S2941	This gene, encoding the p40 subunit of IL-12, drives the differentiation of T lymphocytes into the Th1 subset, which is characterized by the production of cytokines that promote cell-mediated immunity.	T1D: ▪ Morahan *et al *[49] 249 Eastern Australian multiplex families, RR for A allele = 1.40 (1.11–1.77), *P *= 0.0025. 235 Australian simplex families, RR for A allele = 1.84 (1.32–2.56), *P *= 0.00014. ▪ Davoodi-Sermiromi *et al *[41] 364 USA multiplex families, RR for C allele = 1.23 (0.97–1.56), *P *= 0.08. haplotype: allele 2 (D5S2941) and 1159C, *P *= 0.02. ▪ Windsor *et al *[40] 648 cases and 246 controls – Western Australia, OR allele C = 1.6 (1.2–2.0), *P *= 0.001. Excess of heterozygotes in cases ≥ 16 yrs diagnosis, OR = 1.8(1.2–2.7), *P *= 0.005 and ≥ 26 yrs diagnosis, OR = 2.4 (1.5–3.8), *P *= 0.0002. Five studies failed to find an association: ▪ Dahlman *et al *[60] 2,873 families from UK, USA, Finland, Romania ▪ McCormack *et al *[61] 120 cases, 330 controls and 307 families from Northern Ireland ▪ Nistico *et al *[62] 470 cases and 544 controls from Italy. ▪ Bergholdt *et al *[63] 337 Danish simplex and 795 European and American multiplex families. ▪ Johansson *et al *[64] from Norway.

*IL6 *on 7p21 174G>C (rs1800795)	*IL6 *may contribute to the gradual destruction of the pancreatic beta cells, due to its regulatory role in inflammation and the immune response.	T1D: ▪ Kristiansen *et al *[43] 253 Danish families, RR allele C = 1.26 (1.01–1.57), *P*_TDT _= 0.04. Parent-female-trios: RR for C allele = 1.72 (1.25–2.36), *P *= 6.5 × 10^-4^. Parent-male-trios: RR for C allele = 0.91 (0.67–1.24), *P *= 0.58. Age-at-onset males = 14.04 yrs (11.62–16.46) and females = 8.81 yrs (6.60–11.01), interaction model *P *= 0.002. ▪ Gillespie *et al *[44] UK 1,230 cases. *IL6-174CC *less frequent in females diagnosed after, than in those diagnosed before, 10 years, *P *= 0.016. ▪ Jahromi *et al *[65] 257 cases and 120 newborn controls, UK, OR for C allele = 0.54 (0.39–0.74), derived *P *= 7.8 × 10^-5^. 53 parent-child trios RR for C allele = 0.83 (0.48–1.43) ▪ Eerligh *et al *[66] 206 Dutch simplex families, no association.

*ICAM1 *on 19p13 G241R (rs1799969)	*ICAM-1*, upregulated during inflammation and hyperglycemia, is involved in the attachment of lymphocytes to the endothelium as well as in the priming of effector T cells.	T1D: Kristiansen *et al*. [45] 253 Danish families, no association with K469E. Nejentsev *et al *[67] 3,695 families from Finland, UK, USA, Norway & Romania, RR = 0.91 (0.83–0.99) *P *= 0.030. 446 families from Bart's-Oxford study RR = 0.60 (0.40–0.89) *P *= 0.006. Ma *et al*. [68] 432 cases and 187 controls from Sweden. Tested five SNPs, rs281432 and rs5498 had *P*-values of 0.026 and <0.001 respectively when genotype model assumed.

*TBX21 *on 17q21 His33Gln (rs2240017)	*TBX21 *has a role in the complex regulation of T lymphocyte responses, as a master-regulator of Th1 cytokine IFN-γ gene expression.	T1D: ▪ Sasaki *et al *[46], 153 cases and 200 controls from Japan, OR for G allele = 1.77 (1.08–2.91), *P *= 0.02.

*IL23R *on 1p31 Arg381Gln (rs11209026)	The protein encoded pairs with the receptor molecule IL12RB1/IL12Rbeta1, and both are required for IL23A signaling. This protein associates constitutively with Janus kinase 2 (JAK2), and also binds to transcription activator STAT3 in a ligand-dependent manner.	IBD and psoriasis: ▪ Duerr *et al *[69] CD: 547 non-Jewish cases and 548 controls, OR = 0.26 (0.15–0.43), *P *= 5.05 × 10^-9^. 401 Jewish cases and 433 controls, OR = 0.45 (0.27–0.73), *P *= 7.95 × 10^-4^. 883 IBD families, *P *= 1.32 × 10^-10^. ▪ Cargill *et al *[54] Psoriasis: White North American 1446 cases and 1432 controls, OR= 0.63 (0.50–0.79), *P *= 1.89 × 10^-4^.

*IRF5 *on 7q32.1 -3835 (rs2004640)	Member of a family of transcriptional factors that controls inflammatory and immune responses.	SLE: ▪ Sigurdsson *et al *[70], 679 cases and 438 controls from Sweden, Finland and Iceland, *P *= 2.4 × 10^-7^. ▪ Graham *et al *[71] 1,661 cases and 2,508 controls from USA, Spain, Sweden and Argentina, OR = 1.47 (1.36–1.60), *P *= 4.2 × 10^-21^. T1D: One study failed to find an association. Qu *et al *[72] 947 T1D families.

*CD40 *on 20q12 Kozak (rs1883832)	*CD40 *and *CD40L *signalling is known to play an important role in the immune response. The proteins are expressed in a variety of cell types and ligation causes cells to produce inflammatory cytokines and cellular adhesion molecules.	GD: ▪ Tomer *et al *[48] 154 cases and 118 controls – Caucasian, OR for CC genotype = 1.6, *P *= 0.048. ▪ Kim *et al *[73]132 cases and 164 controls from Korea, OR for CC genotype = 1.93 (1.21–3.09), *P *= 0.019. ▪ Mukai *et al *[74] 324 cases and 229 controls from Japan: late onset GD, decrease in TT allele (*P *= 0.011) Two studies failed to find an association: ▪ Houston *et al *[75] 451 cases and 446 controls from UK. ▪ Heward *et al *[76] 800 cases and 785 controls from UK.

T1D = type 1 diabetes, SLE = systemic lupus erythemastosus, RA = Rheumatoid Arthritis, IBD = Inflammatory Bowel disease, CD = Crohn's disease and GD = Graves' disease.

## Methods

### Subjects

Type 1 diabetes families were white European or of white European descent, with two parents and at least one affected child comprising DNA samples from up to 918 Finnish multiplex/simplex families [14], 456 multiplex Diabetes UK Warren I families [15], 278 multiplex Human Biological Data Interchange (HBDI) families [16], 80 Yorkshire simplex families, 263 Belfast multiplex/simplex [17], 360 Norwegian simplex families [18] and 240 Romanian simplex families [19]. The single SNPs from *TAF5L *and *IL12B*, two SNPs from *PDCD1 *and five SNPs from *IL6 *were genotyped in all the families. The *TCF7 *SNP was genotyped in Warren, Yorkshire, Belfast and Romanian families. The *TBX21 *SNP was genotyped in Warren, Yorkshire, HBDI, Belfast and Romanian families. The *CD40 *SNP was genotyped in Warren and HBDI families.

The type 1 diabetes cases (maximum 7,888) [20], the British 1958 Birth Cohort (maximum 8,858) [21] and the UK Blood Services controls (1,500) have been described previously [6,10]. All cases and controls are white European. All DNA samples were collected after approval from the relevant research ethics committees and written informed consent was obtained from the participants.

Additional file 1, Table S1 contains a summary of the samples genotyped for each gene.

### SNP identification and genotyping

*IL12B *is among the genes re-sequenced by the University of Washington: the Fred Hutchinson Cancer Research Centre (UW-FHCRC) Variation Discovery Resource [22]. The *IL12B *SeattleSNPs encompass the introns and exons between 1.7 kb 5' of exon 2 to 2 kb 3' of the untranslated exon 8 of *IL12B*. In this region, they detected 33 polymorphisms in their set of 23 DNA samples from Centre d'Etude du Polymorhisme Humain (CEPH) parents of European descent. As the untranslated exon 1 and 5' region beyond it were not sequenced, we-resequenced, a further 2.9 kb 5', including exon 1 and the known promoter in the same 23 CEPH parents used by SeattleSNPs. This identified a further four polymorphisms including the CTCTAA/CG complex promoter deletion insertion (DIP) (rs17860508), incorrectly described previously as a 4 bp deletion [23].

*IL6 *was also resequenced by the SeattleSNP Project.

SNPs were genotyped using either Taqman MGB chemistry (Applied Biosystems) or Invader Biplex Assay (Third Wave Technologies, Madison). The D5S2941 microsatellite was genotyped using PCR and evaluated size differences using an ABI 3700 capillary sequencer.

### Wellcome Trust Case Control Consortium

We used data from the WTCCC GWA study [10] to determine whether there was any evidence of an association with type 1 diabetes in the LD blocks containing the polymorphisms of interest. LD blocks were defined using Haploview [24] and HapMap Project [25] data for the 60 Centre d'Etude du Polymorphisme Humain (CEPH) parents. We used the four gamete rule [26] for defining LD blocks within Haploview. We note that the 2,000 case and 3,000 (1,500 from the British 1958 Birth Cohort and 1,500 from the UK Blood Services) control samples used by the WTCCC where also genotyped in this study. We required WTCCC SNPs to have a minor allele frequency (MAF) ≥ 0.05, a call rate ≥ 0.99 and no extreme deviation from Hardy-Weinberg equilibrium (HWE) (χ_1_^2 ^≤ 25) [27].

### Statistical Analysis

All statistical analyses were performed in either Stata [28] or R [29] statistical systems. Additional routines may be downloaded [30].

All unaffected parent and control genotypes were assessed for, and found to be in Hardy-Weinberg equilibrium (*P *> 0.05). SNPs genotyped in the family collection were analysed using the transmission/disequilibrium test [31] and, after estimating pseudo-controls [32], conditional logistic regression, respectively modelling allelic relative risks (RRs; a one-degree-of-freedom (df) test) and genotype RRs (a two-df test). In the case and pseudo-control analysis, we consider the transmitted pair of alleles as the "case" and the other three possible pairs of transmitted alleles as "pseudo-controls" in a matched case-control study [32]. The one-df test assumes multiplicative allelic effects and the two-df test assumes no specific mode of inheritance, for example, in the analysis of TCF7 C883A, genotype risks of C/A and A/A were modelled relative to the C/C genotype.

In the case-control collection, we performed similar tests using logistic regression models, stratified by 12 broad geographical regions (Southwestern; Southern; Southeastern; London; Eastern; Wales; Midlands; North Midlands; Northwestern; East and West Riding; Northern; and, Scotland), to allow for geographic variation in allele frequencies across Great Britain [27].

The tag SNPs were selected and analysed using a multilocus test, as previously described [7,33]. Qu *et al*. [8] recently reported that the family-based multilocus test was not confined to heterozygous parents, which compromised the protection against population stratification. This is incorrect, as described in the studies by Chapman *et al*. [33] and Lowe *et al*. [34], only transmissions from heterozygous parents contribute to the test.

## Results

### TAF5L

We genotyped one *TAF5L *SNP (C744A; rs3753886), which has previously been associated with type 1 diabetes [35] (Table 1). We found inconsistent evidence of an association between type 1 diabetes and C744A in the case-control and family collections. In 7,497 case and 7,496 control genotypes, we obtained marginal evidence of an association (*P *= 7.32 × 10^-3^; OR for allele A = 1.07, 95% CI 1.02–1.12; Table 2). Although, in 2,645 parent-child trio genotypes, there was borderline evidence of an association (*P *= 0.0519), the risk for the minor allele (RR for allele A = 0.92, 95% CI 0.85–0.99; Table 3) was going in the opposite direction to that in the case-control collection.

**Table 2:** Association analyses in type 1 diabetes cases and controls.

		Alleles		Genotypes		
*TAF5L*	C744A (rs3753886)	C	A	CC	CA	AA

7,497 cases		7,457 (49.7)	7,537 (50.3)	1,827 (24.4)	3,803 (50.7)	1,867 (24.9)
7,496 controls		7,671 (51.2)	7,321 (48.8)	1,928 (25.7)	3,815 (50.9)	1,753 (23.4)
OR (95% CI)		1.00 (reference)	1.07 (1.02–1.12)	1.00 (reference)	1.05 (0.97–1.14)	1.14 (1.04–1.25)
*P*			7.32 × 10^-3^		0.0244	

*PDCD1*	7146G>A (rs11568821)	G	A	GG	GA	AA

7,888 cases		13,966 (88.3)	1,810 (11.7)	6,196 (78.6)	1,574 (20.0)	118 (1.5)
8,858 controls		15,846 (88.5)	1,870 (11.5)	7,088 (80.0)	1,670 (18.9)	100 (1.1)
OR (95% CI)		1.00 (reference)	1.10 (1.02–1.17)	1.00 (reference)	1.08 (1.00–1.17)	1.32 (1.00–1.74)
*P*			0.0102		0.0268	

*PDCD1*	872C>T (rs2227981)	C	T	CC	CT	TT

5,758 cases		6,699 (58.2)	4,817 (41.8)	1,956 (34.0)	2,787 (48.4)	1,015 (17.6)
7,289 controls		8,440 (57.9)	6,138 (42.1)	2,461 (33.8)	3,518 (48.3)	1,310 (18.0)
OR (95% CI)		1.00 (reference)	1.00 (0.95–1.06)	1.00 (reference)	1.00 (0.92–1.08)	0.97 (0.87–1.07)
*P*			0.586		0.760	

*TCF7*	Pro19Thr (rs5742913)	C	A	CC	CA	AA

7,434 cases		13,022 (87.6)	1,846 (12.4)	5,709 (76.8)	1,604 (21.6)	121 (1.6)
8,637 controls		15,355 (88.9)	1,919 (11.1)	6,826 (79.0)	1,703 (19.7)	108 (1.3)
OR (95% CI)		1.00 (reference)	1.13 (1.06–1.22)	1.00 (reference)	1.13 (1.04–1.22)	1.32 (1.01–1.73)
*P*			4.16 × 10^-4^		1.91 × 10^-3^	

*IL12B*	A1159C (rs3212227)	A	C	AA	AC	CC

4,321 cases		6,898 (79.8)	1,744 (20.2)	2,748 (63.6)	1,402 (32.5)	171 (4.0)
4,711 controls		7,588 (80.5)	1,834 (19.5)	3,050 (64.7)	1,488 (31.6)	173 (3.7)
OR (95% CI)		1.00 (reference)	1.06 (0.98–1.14)	1.00 (reference)	1.06 (0.96–1.16)	1.13 (0.90–1.42)
*P*			0.134		0.324	

*IL6*	174G>C (rs1800795)	G	C	GG	GC	CC

7,785 cases		8,726 (56.0)	6,844 (44.0)	2,928 (33.1)	4,312 (48.7)	1,612 (18.2)
8,852 controls		10,168 (57.4)	7,536 (42.6)	2,456 (31.6)	3,814 (49.0)	1,515 (19.5)
OR (95% CI)		1.00 (reference)	1.05 (1.01–1.10)	1.00 (reference)	1.04 (0.97–1.12)	1.11 (1.01–1.21)
*P*			0.0291		0.0879	

*ICAM1*	G241R (rs1799969)	G	A	GG	GA	AA

5,776 cases		10,257 (88.8)	1,295 (11.2)	4,553 (78.8)	1,151 (19.9)	72 (1.3)
6,094 controls		10,787 (88.5)	1,401 (11.5)	4,787 (78.6)	1,213 (19.9)	94 (1.54)
OR (95% CI)		1.00 (reference)	0.96 (0.89–1.05)	1.00 (reference)	1.00 (0.91–1.10)	0.74 (0.54–1.02)
*P*			0.382		0.179	

*TBX21*	His33Gln (rs2240017)	C	G	CC	CG	GG

4,342 cases		8,456 (97.4)	228 (2.6)	4,116 (94.8)	224 (5.2)	2 (0.1)
4,763 controls		9,293 (97.6)	233 (2.4)	4,533 (95.2)	227 (4.7)	3 (0.1)
OR (95% CI)		1.00 (reference)	1.08 (0.89–1.30)	1.00 (reference)	1.08 (0.88–1.31)	1.10 (0.16–7.54)
*P*			0.459		0.759	

*IL23R*	Arg381Gln (rs11209026)	G	A	GG	GA	AA

6,087 cases		11,413 (93.7)	761 (6.3)	5,343 (87.8)	727 (11.9)	17 (0.3)
6,303 controls		11,776 (93.4)	830 (6.6)	5,499 (87.2)	778 (12.3)	26 (0.4)
OR (95% CI)		1.00 (reference)	0.93 (0.84–1.03)	1.00 (reference)	0.95 (0.85–1.06)	0.67 (0.36–1.26)
*P*			0.183		0.285	

*IRF5*	-3835 (rs2004640)	T	G	TT	TG	GG

5,657 cases		5,834 (51.6)	5,480 (48.4)	1,487 (26.3)	2,860 (50.6)	1,310 (23.2)
6,044 controls		6,160 (51.0)	5,928 (49.0)	1,581 (26.2)	2,998 (49.6)	1,465 (24.2)
OR (95% CI)		1.00 (reference)	0.98 (0.93–1.03)	1.00 (reference)	1.03 (0.94–1.12)	0.96 (0.86–1.06)
*P*			0.451		0.353	

*CD40*	Kozak (rs1883832)	C	T	CC	CT	TT

4,392 cases		6,624 (75.4)	2,160 (24.6)	2,500 (56.9)	1,624 (37.0)	268 (6.1)
4,702 controls		7,132 (75.8)	2,272 (24.2)	2,680 (57.0)	1,772 (37.7)	250 (5.3)
OR (95% CI)		1.00 (reference)	1.03 (0.96–1.10)	1.00 (reference)	0.97 (0.89–1.07)	1.18 (0.97–1.42)
*P*			0.456		0.162	

OR = odds ratio for the minor allele, 95% CI = 95% confidence interval.

**Table 3:** Association analyses in type 1 diabetes families.

				Transmission/disequilibrium test			
				
		Parent-child-trios (families)	Parental MAF	Minor allele		RR (95% CI)	*P*
						
				Transmitted	Untransmitted		
*TAF5L*	A1159C (rs3212227)	2,645 (2,515)	46.8	1,299	1,400	0.92 (0.85–0.99)	0.0519
*PDCD1*	7146G>A (rs11568821)	3,125 (2,742)	9.3	516	538	0.96 (0.85–1.08)	0.498
*PDCD1*	872C>T (rs2227981)	2,190 (1,831)	42.5	1,111	1,043	1.07 (0.98–1.16)	0.143
*TCF7*	C883A (rs5742913)	1,556 (1,224)	11.2	327	314	1.04 (0.89–1.21)	0.608
*IL12B*	A1159C (rs3212227)	3,015 (2,606)	18.9	949	933	1.02 (0.93–1.12)	0.712
*IL6*	174G>C (rs1800795)	2,803 (2,651)	47.0	1,411	1,360	1.04 (0.97–1.12)	0.333
*TBX21*	His33Gln (rs2240017)	1,989 (1,374)	2.7	111	100	1.11 (0.85–1.45)	0.449
*CD40*	Kozak (rs1883832)	1,446 (795)	24.1	522	542	0.96 (0.85–1.08)	0.540

MAF = minor allele frequency, RR = relative risk for minor allele, 95% CI = 95% confidence interval.

We found borderline evidence of an association in the WTCCC, which had two SNPs with a MAF ≥ 0.05 in the 5 kb LD block containing C744A. The lowest *P*-value was for C744A (*P *= 0.0561).

### PDCD1

We genotyped two *PDCD1 *SNPs (7146G>A and 872C>T), which have previously been associated with type 1 diabetes [36] and RA respectively [37] (Table 1). We found inconsistent evidence of an association between type 1 diabetes and 7146G>A in the case-control and family collections (Tables 2 and 3). In 7,888 case and 8,858 control genotypes, we obtained limited evidence of an association (*P *= 0.0102; OR for allele A = 1.10, 95% CI = 1.02–1.17). In 3,125 parent-child trio genotypes, no evidence of an association was found (*P *= 0.498). For the second SNP, 872C>T, no evidence of an association was found in either collection (Tables 2 and 3).

There were no WTCCC SNPs with a MAF ≥ 0.05 in the 12 kb LD block containing 7146G>A. We note that 7146G>A was not included in either HapMap or the WTCCC study.

### TCF7

We genotyped one *TCF7 *SNP (C883A; rs5742913), which had previously been associated with type 1 diabetes [38] (Table 1). Despite obtaining no evidence of an overall association, Noble *et al*. [38] proceeded to analyse subgroups, defined by up to three criteria, in which they found an excess of the A allele transmitted: from fathers; to male offspring; to low HLA risk (non-DR3/DR4) offspring; and, to early onset offspring (Table 1). We found some evidence of an association between type 1 diabetes and C883A in the case-control and family collections (Tables 2 and 3). In 7,434 case and 8,637 control genotypes, in contrast to Noble *et al*. [38], we obtained evidence of an association with type 1 diabetes (*P *= 4.16 × 10^-4^; OR for allele A = 1.13, 95% CI = 1.06–1.22). In 1,556 parent-child trio genotypes, no evidence of an association was found (*P *= 0.608). In addition, we found no evidence of an association between type 1 diabetes and C883A when comparable subgroup analyses, as described by Noble *et al*. [38], were performed (see Additional file 1, Table S2). We also performed, a case-only regression analysis of cases and affected offspring from the case-control and family collections respectively, which showed no consistent evidence of a gender or age-at-diagnosis effect at C883A. Similarly, a case-only gene-gene interaction analysis between C883A and the known type 1 diabetes loci, *HLA, INS, CTLA4 *and *PTPN22*, revealed no consistent evidence of an interaction with C883A (see Additional file 1, Table S3). We note that the lack of evidence for an interaction between C883A and *PTPN22 *1858C>T contradicts a previous study reporting an over-transmission of *PTPN22 *1858T to individuals who have at least one copy of *TCF7 *883A (*P *= 0.015) [39].

We found no evidence of an association in the WTCCC, which had two SNPs with a MAF ≥ 0.05 in the 66 kb LD block containing C883A. The SNP with the lowest *P*-value was rs756699 (*P *= 0.694). We note that C883A was not included in either HapMap or the WTCCC study.

### IL12B

*IL12B *has been reported to be associated with type 1 diabetes in some but not all studies (Table 1). Therefore, we investigated the contribution of *IL12B *to type 1 diabetes susceptibility, as thoroughly as possible, by genotyping a SNP (A1159C; rs3212227), two rare nsSNPs (rs3213096 and rs3213119), a microsatellite (D5S2941) and a set of tag SNPs for the *IL12B *region. In 4,321 case, 4,711 control and 3,015 parent-child genotypes, we obtained no evidence of an association between type 1 diabetes and A1159C (*P *= 0.134 and 0.630 respectively; Tables 2 and 3). In addition, we performed a case-only analysis to replicate the association reported by Windsor *et al*. [40] between A1159C and age-at-diagnosis (Table 1). However, no evidence of an age-at-diagnosis effect at A1159C was found (see Additional file 1, Table S4).

The microsatellite D5S2941, in *IL12B *intron 2, had previously been reported to be associated with type 1 diabetes by Davoodi-Semiromi *et al*. [41] (*P *< 0.006; Table 1). They detected two alleles, a major allele 1 with eight repeat units and a minor allele 2 with nine repeat units, and found over-transmission of allele 2 to affected offspring. We detected an additional two, extremely rare, alleles: allele 3 (ten repeat units) and allele 4 (seven repeat units), both found at frequency of <0.001. In 1,327 case and 1,160 control genotypes, we obtained no evidence of an association between type 1 diabetes and D5S2941 allele 2 (*P *= 0.114). Davoodi-Semiromi *et al*. also reported an association between type 1 diabetes and the D5S2941 allele 2-1159C haplotype (*P *= 0.02) and suggested the possibility that the causal variant remained ungenotyped and elsewhere in *IL12B *[41]. Consequently, we also analysed this haplotype using an EM algorithm-based routine to assign haplotypes to cases and controls, which were then analysed using a linear model weighted by the posterior haplotype probabilities for each case or control. Again we found no association with disease for this haplotype in 1,298 case and 1,111 control genotypes (*P *= 0.195).

To investigate the possibility of a polymorphism associated with type 1 diabetes in *IL12B *that we, or others, have not yet genotyped, we adopted an linkage disequilibrium (LD) mapping approach using tag SNPs (Table 4). We combined resequencing data in 23 CEPH parents from the SeattleSNP project [22] with inhouse resequencing of the untranslated exon 1 and 5', not resequenced in the SeattleSNP project (Methods). In the combined resequencing data, we identified 38 polymorphisms, comprising 34 SNPs (four SNPs provided by the in house resequencing), three deletion-insertion polymorphisms (DIPs) and the microsatellite D5S2941 (see Additional file 1, Table S5). Six tag SNPs were selected (minimum *R*^2 ^= 0.80) from 25 SNPs with a minor allele frequency (MAF) ≥ 0.10. The set of tag SNPs was genotyped in the case-control collection and analysed using a multilocus test, which tests for an association between type 1 diabetes and the tag SNPs due to LD with one or more causal variants [33]. The multilocus test *P-*value was 0.940, providing no evidence of an association between type 1 diabetes and the *IL12B *region (Table 4). We note that rs17860508, the promoter DIP CTCTAA/CG, was selected as a tag SNP. Previously, this polymorphism had been associated with asthma susceptibility [23] and IgE levels [42], but we found no association with type 1 diabetes (4,367 case and 4,714 control genotypes, *P *= 0.878).

**Table 4:** Tag SNP results for *IL6 *and *IL12B *regions.

					Multilocus test *P*-values	
					
	Exons	Sequencing source	Polyorphisms	Tag SNPs	Families	Cases and controls
*IL12B*	8	Seattle SNPs and in house (23 subjects)	33 (from Seattle) 4 (in house) 25 had a MAF ≥ 0.10	6	N/A	0.940 1,590 cases and 1,748 controls
*IL6*	6	Seattle SNPs (23 subjects)	49 12 had MAF ≥ 0.10	4	0.0231 3,320 parent-child trios	0.236 3,486 cases and 3,783 controls

We selected tag SNPs from SNPs with a MAF ≥ 0.10. MAF = minor allele frequency.

Finally, we tested for an association between type 1 diabetes and two rare nsSNPs (rs3213096 and rs3213119). Although the MAF was 0.022 for both nsSNPs in the SeattleSNPs CEPH sequencing panel, in the case-control collection, rs321096 had a much lower MAF of 0.0035 in controls and consequently, we had no power in a collection of 4,383 case and 4,732 control genotypes to test for an association. The other nsSNP, rs3213199, with MAF of 0.034 in controls, showed no evidence of an association with type 1 diabetes in 4,348 case and 4,691 control genotypes (*P *= 0.0941).

We found limited evidence of an association in the WTCCC, which had six SNPs with a MAF ≥ 0.05 in the 14 kb LD block containing A1159C. The only associated SNP was rs6859018 (*P *= 0.0274), which is in perfect LD (r^2 ^= 1) with A1159C in 60 CEPH parents. Consequently, based on the perfect LD between rs6859018 and A1159C, we can conclude that this association is a false positive. We note that A1159C was not included in the WTCCC study.

### IL6

As *IL6 *has been reported to be associated with type 1 diabetes [43] (Table 1), we sought to replicate this association by genotyping *IL6-174G>C *(rs1800795) and a set of tag SNPs for the *IL6 *region. We found inconsistent evidence of an association between type 1 diabetes and *IL6-174G>C *in the case-control and family collections (Tables 2 and 3). In 7,785 case and 8,852 control genotypes, we obtained limited evidence of an association with type 1 diabetes (*P *= 0.0291; OR for allele C = 1.05, 95% CI = 1.01–1.10). In 2,803 parent-child trio genotypes, no evidence of an association was found (*P *= 0.333). As Kristiansen *et al*. [43] found evidence that the IL6-174C allele was only associated with type 1 diabetes in female offspring (Table 1), we analysed *IL6-174G>C *by sex. In the cases and controls, we obtained limited evidence of an association in males (*P *= 0.0378), but not in females (see Additional file 1, Table S6a) and in the families, we found no evidence of an association in either male or female type 1 diabetes offspring (see Additional file 1, Table S6b).

Previously, Gillespie *et al*. [44] found frequency differences in *IL6-174G>C *genotypes between males and females diagnosed ages > 10 years. Consequently, we conducted a similar case-only analysis using a multinomial logistic regression model to adjust for broad geographical region within Great Britain and for population of the cases and affected offspring, respectively. We found no evidence of genotype differences between 5,700 male and 5,292 female cases and affected offspring (*P *= 0.370) and when analysed by age-at-diagnosis (see Additional file 1, Table S7).

To test for an association between type 1 diabetes and the *IL6 *region, we adopted a LD mapping approach. We used SeattleSNP resequencing data in 23 CEPH parents, four tag SNPs were selected (minimum *R*^2 ^= 0.80) from twelve SNPs with a MAF ≥ 0.10 (see Additional file 1, Table S8). We found no evidence for association in the case-control collection (multilocus test *P *= 0.236). However, in the family collection, limited evidence of an association was found (multilocus test *P *= 0.0231) (Table 4).

We found no evidence of an association in the WTCCC, which had two SNPs with a MAF ≥ 0.05 in the 4 kb LD block containing *IL6-174G>C*. The SNP with the lowest *P*-value was rs2069835 (*P *= 0.343). We note that *IL6-174G>C *was included in the WTCCC study, but was dropped as the call rate was below 0.99.

### ICAM1

We genotyped one *ICAM1 *SNP (G241R; rs1799969), which has previously been associated with type 1 diabetes [45] (Table 1). However, no evidence of an association was found in 5,776 cases and 6,094 controls (*P *= 0.382; Table 2).

We found some evidence of an association in the WTCCC, which had two SNPs with a MAF ≥ 0.05 in the 15 kb LD block containing G241R. The most associated SNP was rs892188 (*P *= 0.0257; OR for allele T = 1.10, 95% CI = 1.01–1.19), located just over 2 kb upstream of the 3' UTR of *ICAM5*, which has low LD (r^2 ^= 0.274) with G241R. We note that G241R was not included in the WTCCC study.

### TBX21

We genotyped one *TBX21 *SNP (His33Gln; rs2240017), which had previously been associated with type 1 diabetes in a Japanese case and control collection [46] (Table 1). No evidence for association in either collection was found (Tables 2 and 3). We note that the G (Gln) allele frequency in controls was 0.024 (Table 2) and in parents 0.027, considerably lower than reported in Japanese by Sasaki *et al*. (MAF = 0.105) [46], but similar to that found in other European populations (MAF = 0.030) [47].

We found no evidence of an association in the WTCCC, which had one SNP with a MAF ≥ 0.05 in the 14 kb LD block containing His33Gln. The SNP, rs2240017, had a *P*-value of 0.131. We note that His33Gln was not included in the WTCCC study.

### IL23R

We genotyped one *IL23R *SNP (Arg381Gln; rs11209026), which has previously been associated with IBD and psoriasis (Table 1). In 6,087 cases and 6,303 controls, we found no evidence of an association with type 1 diabetes (*P *= 0.183; Table 2).

The only WTCCC SNP with a MAF ≥ 0.05 in a 15 kb LD block containing Arg381Gln was Arg381Gln (*P *= 0.857).

### IRF5 and CD40

We genotyped two SNPs from *IRF5 *and *CD40 *(-3835/rs2004640 and 168A>G/rs1883832, respectively), which have previously been associated with SLE and GD respectively (Table 1). The *CD40 *-168A>G SNP has been reported to disrupt the Kozak consensus sequence necessary for efficient translation [48]. No evidence of an association was found for either SNP (Tables 2 and 3).

We found no evidence of an association in the WTCCC, which had no SNPs with a MAF ≥ 0.05 in the 5 kb LD block containing -3835 and 168A>G was not contained within a block. Neither -3835 nor 168A>G were included in the WTCCC study.

## Discussion

In this study, we have tested ten candidate genes for an association with type 1 diabetes using large case-control and family collections. We did obtain some evidence, albeit inconsistent between collections, of an association with *TAF5L *(C744A), *PDCD1 *(7146G>A), *TCF7 *(C883A) and *IL6 *(*IL6-174G>C*, rs2069849 and the *IL6 *region). Although *TAF5L *(C744A; rs3753886), *PDCD1 *(7146G>A; rs11568821), *TCF7 *(C883A; rs5742913) and *IL6 *(*IL6-174G>C*; rs1800795) have previously been associated with type 1 diabetes, the possibility remains that these associations are false positives. However, the findings reported here maybe the result of the case-control collection having more power than the family collection to detect SNPs with relatively small effects in type 1 diabetes or being in weak LD with the causal locus. Consequently, additional studies will be required to ascertain the contribution of *TAF5L*, *PDCD1*, *TCF7 *and *IL6 *to type 1 diabetes susceptibility. The case-control collection (8,000 cases and 8,000 controls) provided about 60% power to detect an OR of 1.2 for a MAF of 0.10 at a *P*-value of about 1 × 10^-6^; and about 96% power for a MAF of 0.20. The family collection (3,125 parent-child trios) provided less power to detect an OR of 1.2, after increasing the *P*-value to 1 × 10^-3^, there was about 45.1 % power for a MAF of 0.10 and about 81.4% power for a MAF of 0.20.

We did not obtain any evidence of an association between type 1 diabetes and either *ICAM1*, *IL12B *or *TBX21*, all of which had previously been associated with type 1 diabetes [45,46,49]. However, limited evidence of an association with rs892188, located in the LD block containing the reported SNP in *ICAM1*, was provided by the WTCCC. Additional studies will be required to ascertain the contribution of rs892188 to type 1 diabetes susceptibility. The previous reports of disease associations may well have been false positives, which have to be expected given: the low prior probability, even for candidate genes [1,50], of detecting a true causal locus of complex disease; the frequent use of relatively small sample sizes and of nominal levels of significance; and, the large numbers of SNPs tested for an association with type 1 diabetes. It is interesting to note that when, by chance, a true positive result is found, as in the case for *PTPN22 *Arg620Trp SNP in type 1 diabetes [5], it is replicated by many groups, very rapidly [6,39,51,52], although this has a large effect approaching an OR = 2.

Finally, we did not obtain any evidence of an association between type 1 diabetes and *IL23R*, *IRF5 *or *CD40*, all of which have previously been associated with other immune-mediated diseases including, most recently, *IL23R *in Crohn's disease[10,53] and in psoriasis [54]. Lack of association of *IL23R*, *IRF5 *and *CD40 *with type 1 diabetes helps to delineate pathogenic mechanisms between type 1 diabetes and other immune-mediated diseases, especially when the associations reported for the other diseases are highly likely to be true positive results. Nevertheless, for these and other loci, it is possible that one or more of these genes could have allelic heterogeneity in which one allele predisposes to certain autoimmune diseases and a second allele at a different location in the gene predisposes to another. This possibility necessitates the continued investigation of further polymorphisms within each gene.

## Conclusion

The functional candidate gene approach has now been superseded by GWA studies, which are detecting major susceptibility loci [10,11,53,55]. Most of the confirmed loci from GWA studies have ORs ≤ 1.3, consistent with an L-shaped distribution of allelic effect sizes (that is, a small number of genes with large effects and a large number of genes with small effects) [1,11]. *TAF5L*, *PDCD1*, *TCF7*, *IL6 *and *ICAM1 *may be amongst the numerous loci with small effects in type 1 diabetes. We note that genes found with small effects on disease may have much larger effects in subgroups of phenotypically defined cases. For example, *CTLA4 *genotypes has a small effect overall in type 1 diabetes (OR = 1.20, 95% CI 1.13–1.27), but subclassification of cases with or without the thyroid peroxidase autoantibodies revealed an increased effect (OR = 1.49, 95% CI 1.29–1.72) for cases with autoantibodies (without autoantibodies OR = 1.16, 95% CI 1.10–1.24) [56].

## Competing interests

The author(s) declare that they have no competing interests.

## Authors' contributions

JDC contributed to data analysis and drafted the manuscript. DJS drafted the manuscript and worked on *IL23R*, *IRF5 *and *CD40 *studies. RB drafted the manuscript and worked on *TCF7 *and *TBX21 *studies. FP drafted the manuscript and worked on the *IL12B *study. KD worked on the *TAF5L *study. LMG drafted the manuscript and worked on the *IL6 *study. JM drafted the manuscript and worked on the *PDCD1 *study. LRZ worked on *PDCD1*, *TCF7 *and *ICAM1 *studies. AV drafted the manuscript and worked on the *TCF7 *study. NMW managed the data. JAT participated in the conception, design and coordination of the studies, data analysis and drafted the manuscript. All authors read and approved the final manuscript.

## Pre-publication history

The pre-publication history for this paper can be accessed here:



## Supplementary Material

Additional file 1 Supplementary Tables. Summary tables of additional analyses conducted. Click here for file
