# Supplementary material for: The candidate genes TAF5L, TCF7, PDCD1, IL6 and ICAM1 cannot be excluded from having effects in type 1 diabetes
Source: BMC Med Genet. 2007 Nov 28;8:71. doi: 10.1186/1471-2350-8-71 (PMC2217539; doi:10.1186/1471-2350-8-71)
Supplement: Additional file 1 — Supplementary Tables. Summary tables of additional analyses conducted. [file 1471-2350-8-71-S1.doc]

| Gene and polymorphism | Samples analysed |
| --- | --- |
| *TAF5L* on 1q42  C744A (rs3753886) | 7,497 case and 7,496 control genotypes  2,645 parent-child trio genotypes from 2,515 families (709 Finnish, 469 UK Warren, 360 Norwegian, 331 HBDI, 307 Romanian, 259 Belfast and 80 Yorkshire families) |
| *PDCD1* on 2q37  7146G>A (rs11568821)  872C>T (rs2227981) | 7,888 case and 8,858 control genotypes  3,125 parent-child trio genotypes from 2,742 families (827 Finnish, 469 UK Warren, 412 Romanian, 360 Norwegian, 331 HBDI, 263 Belfast and 80 Yorkshire families)  5,758 case and 7,289 control genotypes  2,190 parent-child trio genotypes from 1,831 families (468 UK Warren, 363 Romanian, 333 Norwegian, 331 HBDI, 256 Belfast and 80 Yorkshire families) |
| *TCF7* on 5q31  Pro19Thr (rs5742913) | 7,434 case and 8,637 control genotypes  1,556 parent-child trio genotypes from 1,224 families (470 UK Warren, 411 Romanian, 263 Belfast and 80 Yorkshire families) |

| *IL12B* on 5q33.3  A1159C (rs3212227)  Microsatellite D5S2941  six tag SNPs  rs321096  rs3213199 | 4,321 case and 4,7116 control genotypes  3,015 parent-child trio genotypes from 2,606 families (873 Finnish, 468 UK Warren, 359 Norwegian, 331 HBDI, 263 Romanian, 232 Belfast and 80 Yorkshire families)  1,327 case and 1,160 control genotypes  1,590 case and 1,748 control genotypes  4,383 case and 4,732 control genotypes  4,348 case and 4,691 control genotypes |
| --- | --- |
| *IL6* on 7p21  174G>C (rs1800795)  four tag SNPs | 7,785 case and 8,852 control genotypes  2,803 parent-child trio genotypes from 2,651 families (916 Finnish, 468 UK Warren, 360 Norwegian, 331 HBDI, 263 Belfast, 233 Romanian and 80 Yorkshire families)  3,486 case and 3,783 control genotypes  3,320 parent-child trio genotypes from 2,675 families (918 Finnish, 469 UK Warren, 360 Norwegian, 331 HBDI, 263 Belfast, 254 Romanian and 80 Yorkshire families) |

| *ICAM1* on 19p13  G241R (rs1799969) | 5,776 case and 6,094 control genotypes |
| --- | --- |
| *TBX21* on 17q21  His33Gln (rs2240017) | 4,342 case and 4,763 control genotypes  1,989 parent-child trio genotypes from 1,374 families (468 UK Warren, 330 HBDI, 263 Belfast, 233 Romanian and 80 Yorkshire families) |
| *IL23R* on 1p31  Arg381Gln (rs11209026) | 6,087 case and 6,303 control genotypes |
| *IRF5* on 7q32.1  -3835 (rs2004640) | 5,657 case and 6,044 control genotypes |
| *CD40* on 20q12  Kozak (rs1883832) | 4,392 case and 4,702 control genotypes  1,446 parent-child trio genotypes from 795 families (464 UK Warren and 331 HBDI families) |

Table S1. A summary of the samples genotyped for each gene.

| Transmissions | Number of parent-child trio genotypes | Transmission/disequilibrium test | |
| --- | --- | --- | --- |
|  |  | Allele A [transmitted:untransmitted (RR)] | P |
| All families | 1,556 | 327:314 (1.04) | 0.608 |
| Subgroup defined by single criterion |  |  |  |
| From father | 778 | 158:136 (1.16) | 0.199 |
| From mother | 778 | 133:142 (0.94) | 0.587 |
| To male offspring | 798 | 175:153 (1.14) | 0.224 |
| To female offspring | 761 | 151:159 (0.95) | 0.650 |
| To early-onset offspring | 806 | 185:159 (1.16) | 0.161 |
| To late-onset offspring | 706 | 133:146 (0.91) | 0.436 |
| To low-HLA risk offspring | 867 | 179:168 (1.07) | 0.555 |
| To high-HLA risk offspring | 565 | 121:124 (0.98) | 0.848 |
| Subgroup defined by two criterion |  |  |  |
| To low-HLA risk offspring from father | 434 | 87:76 (1.14) | 0.389 |
| To low-HLA risk offspring from mother | 433 | 70:70 (1.00) | 1.000 |
| To high-HLA risk offspring from father | 283 | 57:51 (1.12) | 0.564 |
| To high-HLA risk offspring from mother | 282 | 52:61 (0.85) | 0.397 |

Table S2. Subgroup association analysis of *TCF7* SNP C883A in 1,556 parent-child trios. The data were divided into two groups according to age of diagnosis as defined by Noble *et al*. [1], early age of onset was defined as onset of type 1 diabetes at or before 10 years and late age of onset is after 10 years. The data were also divided into two groups according to HLA risk as defined by Noble *et al* [1], a high risk group, with either an HLA-DR3 or –DR4 haplotype (including only DRB1*0401, DRB1*0402, DRB1*0404 and DRB1*0405 haplotypes carrying DQB1*0302 or DQB1*02) and the second group is low risk with neither HLA-DR3 or –DR4. RR = relative risk for minor allele.

|  | Affected offspring  *P* (*n* individuals) | Cases  *P* (*n* individuals) |
| --- | --- | --- |
| Sex | 0.259 (1,682) | 0.0271 (7,433) |
| Age at diagnosis (early/late) | 0.475 (1,625) | 0.818 (7,425) |
| *HLA-DRB1* (low/high risk) | 0.666 (1,537) | 0.381 (1,807) |
| *INS* VNTR | 0.833 (1,540) | 0.0950 (4,015) |
| *PTPN22* 1858C>T | 0.681 (1,423) | 0.382 (5,104) |
| *CTLA4* (CT60) | 0.228 (1,522) | 0.855 (3,982) |

Table S3. Case-only regression analysis of *TCF7* SNP C883A. The data were divided into two groups according to age of diagnosis as defined by Noble *et al*. [1], early age of onset was defined as onset of type 1 diabetes at or before 10 years and late age of onset is after 10 years. The data were also divided into two groups according to HLA risk as defined by Noble *et al* [1], a high risk group, with either an HLA-DR3 or –DR4 haplotype (including only DRB1*0401, DRB1*0402, DRB1*0404 and DRB1*0405 haplotypes carrying DQB1*0302 or DQB1*02) and the second group is low risk with neither HLA-DR3 or –DR4.

|  | Genotypes | | | Alleles | |
| --- | --- | --- | --- | --- | --- |
|  | AA | AC | CC | A | C |
| Cases < 16 years | 4,627 (64.3) | 2,290 (31.8) | 275 ( 3.8) | 11,544 (80.3) | 2,840 (19.7) |
| Cases ≥ 16 years | 224 (63.1) | 115 (32.4) | 16 ( 4.5) | 563 (79.3) | 147 (20.7) |
| Cases ≥ 26 years | 46 (68.7) | 19 (28.4) | 2 ( 3.0) | 111 (82.8) | 23 (17.2) |
|  | Number of cases | OR for C allele (95% CI) | P | OR for heterozygotes^†^ (95% CI) | P |
| Cases < 16 years | 7,192 |  |  |  |  |
| Cases ≥ 16 years | 355 | 1.02 (0.83-1.24) | 0.876 | 0.99 (0.77-1.26) | 0.913 |
| Cases < 26 years | 7,480 |  |  |  |  |
| Cases ≥ 26 years | 67 | 0.80 (0.51-1.27) | 0.329 | 0.79 (0.46-1.36) | 0.390 |

Table S4. *IL12B* A1159C allele and genotype frequencies and subgroup analysis by age-at-diagnosis in 4,311 cases and 3,236 affected offspring. The age-at-diagnosis subgroups were defined as in Windsor *et al.* [2]. Odds ratios (ORs) for cases older at diagnosis versus cases younger at diagnosis. ^†^OR for heterozygotes relative to AA.

| dbSNP | SeattleSNP Name | Map Position (NCBI34) | Major Allele | Minor Allele Frequency | Min *R*² | Location |
| --- | --- | --- | --- | --- | --- | --- |
|
| **rs17860508** | **-** | **158741094-9** | **CG** | **0.472** | **tag SNP** | **Promoter** |
| **rs2546890** | **-** | **158740795** | **T** | **0.435** | **tag SNP** | **5'** |
| rs17860510 | - | 158740426 | G | 0.045 | - | 5' |
| rs17860509 | - | 158740412 | G | 0.023 | - | 5' |
| **rs3213086** | **IL12B-000136** | **158736243-5** | **GT** | **0.217** | **tag SNP** | **intron 1** |
| rs3212217 | IL12B-000356 | 158736025 | G | 0.217 | 100 | intron 1 |
| rs3212218 | IL12B-000890 | 158735491 | G | 0.214 | 94 | intron 1 |
| rs3213117 | IL12B-001018 | 158735363 | A | 0.025 | - | intron 1 |
| rs3212219 | IL12B-001025 | 158735356 | G | 0.200 | 94 | intron 1 |
| rs3212220 | IL12B-001291 | 158735090 | G | 0.196 | 94 | intron 1 |
| **rs3181216** | **IL12B-002508** | **158733873** | **T** | **0.316** | **tag SNP** | **intron 2** |
| rs3181217 | IL12B-003216 | 158733165 | G | 0.190 | 94 | intron 2 |
| rs3181218 | IL12B-003340 | 158733041 | G | 0.190 | 95 | intron 2 |
| rs3181219 | IL12B-003347 | 158733034 | G | 0.068 | - | intron 2 |
| rs3213118 | IL12B-004010 | 158732371 | G | 0.023 | - | intron 2 |
| rs2569254 | IL12B-004237 | 158732144 | G | 0.239 | 100 | intron 2 |
| D5S2941^1^ | - | 158731999-2022 | allele 1 | a | microsatellite | intron 2 |
| rs2569253 | IL12B-004496 | 158731888 | G | 0.413 | 89 | intron 2 |
| rs3213093 | IL12B-004510 | 158731874 | G | 0.174 | 97 | intron 2 |
| rs3213094 | IL12B-004721 | 158731664 | G | 0.182 | 97 | intron 2 |
| rs3213096^2^ | IL12B-005161 | 158731224 | G | 0.023 | - | exon 2 |
| rs2288831 | IL12B-005477 | 158730908 | A | 0.205 | 94 | intron 3 |
| rs2853694 | IL12B-006402 | 158729983 | A/C | 0.500 | 91 | intron 4 |
| rs3213097 | IL12B-006811 | 158729574 | T | 0.196 | 94 | intron 4 |
| rs919766 | IL12B-007926 | 158728459 | T | 0.065 | **-** | intron 4 |
| rs3213102 | IL12B-008894 | 158727491 | G | 0.024 | - | intron 4 |
| **rs2421047** | **IL12B-009183** | **158727202** | **C** | **0.184** | **tag SNP** | **intron 5** |
| rs2853696 | IL12B-010830 | 158725555 | G | 0.182 | 83 | intron 6 |
| rs2195940 | IL12B-011138 | 158725247 | G | 0.065 | - | intron 6 |
| rs3213119^2^ | IL12B-011702 | 158724683 | G | 0.024 | - | exon 6 |
| **rs2853697** | **IL12B-012087** | **158724298** | **A** | **0.239** | **tag SNP** | **intron 7** |
| rs2853697 | IL12B-012258 | 158724126-7 | del | 0.205 | 94 | intron 7 |
| rs3213120 | IL12B-012383 | 158724003 | G | 0.026 | - | 3'UTR |
| rs3212227^3^ | IL12B-012542 | 158723845 | A | 0.196 | 94 | 3'UTR |
| rs1368439 | IL12B-013478 | 158722909 | A | 0.159 | 83 | 3'UTR |
| rs3181224 | IL12B-014642 | 158721745 | T | 0.068 | - | 3' |
| rs3181225 | IL12B-014869 | 158721518 | C | 0.239 | 100 | 3' |
| rs3181226 | IL12B-014962 | 158721425 | G | 0.250 | 100 | 3' |

Table S5. Polymorphisms identified in *IL12B*, showing the minor allele frequency, tag SNPs chosen and the minimum R^2^ value [3]for the SNPs with a MAF ≥ 0.10 that were not selected as tag SNPs. Six tag SNPs (shown in boldface) were selected (minimum R^2^ = 0.80) from 25 SNPs with MAF ≥ 0.10. Map positions on human chromosome 5 from NCBI build 34. Minor allele frequencies shown are based on the SeattleSNPs database from 23 CEPH parent DNAs. ^1^Denotes the (ATT)_n_ microsatellite in intron 2. This microsatellite has four alleles: the major allele of eight repeats (allele 1, 0.78 in controls), nine repeats (allele 2, 0.22 in controls), ten repeats (allele 3, < 0.001 in controls) and seven repeats (allele 4, MAF < 0.001 in controls). ^2^Denotes a non-synonymous SNP (rs3213096, Val>Ile; rs3213119, Val>Phe). ^3^Denotes the SNP C1159A.

|  |  |  | Logistic regression | | |
| --- | --- | --- | --- | --- | --- |
| Male | Cases (%) | Controls (%) | OR | 95% CI | *P* |
| 4,043 case and 4,462 control genotypes | | | | | |
| G | 4,499 (55.6) | 5,134 (57.5) | 1.00 | (reference) |  |
| C | 3,587 (44.4) | 3,790 (42.5) | 1.07 | 1.00 – 1.14 | 0.0378 |
| Genotypes |  |  |  |  |  |
| GG | 1,276 (31.6) | 1,476 (33.1) | 1.00 | (reference) |  |
| CG | 1,947 (48.2) | 2,182 (48.9) | 1.01 | 0.91 – 1.11 | 0.0383 |
| CC | 820 (20.3) | 804 (18.0) | 1.16 | 1.02– 1.31 |
| Female | Cases (%) | Controls (%) | OR | 95% CI | *P* |
| 3,762 case and 4,497 control genotypes | | | | | |
| G | 4,247 (56.4) | 5,146 (57.2) | 1.00 | (reference) |  |
| C | 3,277 (43.6) | 3,848 (42.8) | 1.03 | 0.97 – 1.10 | 0.346 |
| Genotypes |  |  |  |  |  |
| GG | 1,182 (31.4) | 1,472 (32.7) | 1.00 | (reference) |  |
| CG | 1,883 (50.1) | 2,202 (49.0) | 1.07 | 0.97 – 1.18 | 0.439 |
| CC | 697 (18.5) | 823 (18.3) | 1.05 | 0.92 – 1.20 |

Table S6a*. IL6-174G>C* allele and genotype frequencies and subgroup analysis by sex results in the case-control collection. In the analysis of both males and females, we assumed the multiplicative models (males: χ_1_^2^ = 5.59 and *P* = 0.018. females: χ_1_^2^ = 0.89 and *P* = 0.346), as the models were not significantly different (males: χ_1_^2^ = 4.31 and *P* = 0.0378. females: χ_1_^2^ = 0.76 and *P* = 0.384) from the full genotype model (males: χ_2_^2^ = 6.53 and *P* = 0.0383. females: χ_2_^2^ = 1.65 and *P* = 0.439 ). OR = Odds ratio for minor allele, 95% CI = 95% confidence interval.

| Transmission/disequilibrium test | | | | | |
| --- | --- | --- | --- | --- | --- |
| Male  (1,454 trios) | Transmitted | Untransmitted | RR | 95% CI | *P* |
| Allele C | 715 | 692 | 1.03 | 0.93-1.14 | 0.540 |
| Genotypes |  | | Conditional logistic regression | | |
| GG | 417 (28.7) | 1,269 (29.1) | 1.00 | (reference) |  |
| CG | 714 (49.1) | 2,152 (49.3) | 1.02 | 0.88-1.19 | 0.813 |
| CC | 323 (22.2) | 941 (21.6) | 1.07 | 0.87-1.32 |
| Female (1,347 trios) |  |  |  |  |  |
| Allele C | 694 | 667 | 1.04 | 0.94-1.16 | 0.464 |
| Genotypes |  | | Conditional logistic regression | | |
| GG | 386 (28.7) | 1,149 (28.4) | 1.00 | (reference) |  |
| CG | 654 (48.6) | 2,034 (50.3) | 0.97 | 0.83-1.13 | 0.380 |
| CC | 3074 (22.8) | 858 (21.2) | 1.09 | 0.88-1.35 |

Table S6b*. IL6-174G>C* allele and genotype frequencies and subgroup analysis by sex in the family collection. RR = Relative risk for minor allele, 95% CI = 95% confidence interval. ^†^Untransmitted (pseudo-controls) genotypes estimated as in [4].

|  | Females | Males | *P* |
| --- | --- | --- | --- |
| Number of subjects | 5,279 | 5,696 |  |
| Mean age-at-diagnosis | 8.06 years | 8.20 years |  |
| G/G | 1,613 (30.6 %) | 1,751 (30.7 %) |  |
| G/C | 2,615 (49.5 %) | 2,753 (48.3 %) |  |
| C/C | 1,051 (19.9 %) | 1,192 (20.9 %) | 0.359 |
|  |  |  |  |
| Diagnosed ≤ 10 years of age |  |  |  |
| Number of subjects | 3,704 | 3,827 |  |
| Mean age-at-diagnosis | 5.87 years | 5.60 years |  |
| G/G | 1,129 (30.5 %) | 1,185 (31.0 %) |  |
| G/C | 1,833 (49.5 %) | 1,837 (48.0 %) |  |
| C/C | 742 (20.3 %) | 805 (21.0 %) | 0.394 |
|  |  |  |  |
| Diagnosed > 10 years of age |  |  |  |
| Number of subjects | 1,575 | 1,869 |  |
| Mean age-at-diagnosis | 13.21 years | 13.52 years |  |
| G/G | 484 (30.7 %) | 566 (30.3 %) |  |
| G/C | 782 (49.7 %) | 916(49.0 %) |  |
| C/C | 309 (19.6 %) | 387 (20.7 %) | 0.765 |

Table S7. The subgroup analysis of the *IL6-174G>C* polymorphism in 7,795 cases and 3,180 affected offspring. The age-at-diagnosis subgroups were defined as in Gillespie *et al.* [5].

| dbSNP ID | Map Position | Location | MAF | R^2^ |
| --- | --- | --- | --- | --- |
| rs2069824 | 22538472 | 5'UTR | 0.02 | - |
| **rs2069825** | **22538581** | **5'UTR** | **0.46** | **tag SNP** |
| **rs2069827** | **22538696** | **5'UTR** | **0.11** | **tag SNP** |
| rs1800797 | 22539461 | Promoter | 0.48 | 83.24 |
| rs1800795 | 22539885 | Promoter | 0.50 | 80.17 |
| rs2069857 | 22540074 | Promoter | 0.02 | - |
| rs2069858 | 22540565 | Intron | 0.02 | - |
| rs2069832 | 22540673 | Intron | 0.50 | 80.17 |
| rs2069833 | 22540904 | Intron | 0.50 | 80.17 |
| rs1474348 | 22541148 | Intron | 0.50 | 80.17 |
| rs2069837 | 22541267 | Intron | 0.04 | - |
| rs1474347 | 22541364 | Intron | 0.50 | 80.17 |
| rs2069840 | 22541812 | Intron | 0.37 | 80.62 |
| rs1554606 | 22541947 | Intron | 0.43 | 92.54 |
| rs1548216 | 22543013 | Intron | 0.07 | - |
| rs2069843 | 22543234 | Intron | 0.07 | - |
| rs2069844 | 22543250 | Intron | 0.02 | - |
| **rs2069845** | **22543389** | **Intron** | **0.45** | **tag SNP** |
| rs2069860 | 22544278 | Exon 5 | 0.04 | - |
| rs2069849 | 22544396 | Exon 5 | 0.07 | - |
| rs2069861 | 22544894 | 3'UTR | 0.05 | - |
| rs2069862 | 22545855 | 3'UTR | 0.02 | - |
| **rs1818879** | **22545967** | **3'UTR** | **0.29** | **tag SNP** |

Table S8. *IL6*: map positions, locations, minor allele frequency, tag SNPs chosen and the minimum R^2^ values [3] for SNPs with a MAF ≥ 0.10 not selected as tag SNPs. Four tag SNPs (shown in boldface) were selected (minimum R^2^ = 0.80) from twelve SNPs with a MAF ≥ 0.10. All SNPs in *IL6* from Seattle SNP database, MAF= minor allele frequency based on 23 control individuals, map positions based on NCBI build 35, R^2^ values for tagging of SNP.

References

1. Noble JA, White AM, Lazzeroni LC, Valdes AM, Mirel DB, Reynolds R, Grupe A, Aud D, Peltz G, Erlich HA: **A polymorphism in the TCF7 gene, C883A, is associated with type 1 diabetes**. *Diabetes* 2003, **52**:1579-1582.

2. Windsor L, Morahan G, Huang D, McCann V, Jones T, James I, Christiansen FT, Price P: **Alleles of the *IL12B* 3'UTR associate with late onset of type 1 diabetes**. *Hum Immunol* 2004, **65**:1432-1436.

3. Chapman JM, Cooper JD, Todd JA, Clayton DG: **Detecting disease associations due to linkage disequilibrium using haplotype tags: a class of tests and the determinants of statistical power**. *Hum Hered* 2003, **56**:18-31.

4. Cordell HJ, Clayton DG: **A unified stepwise regression procedure for evaluating the relative effects of polymorphisms within a gene using case/control or family data: application to HLA in type 1 diabetes**. *Am J Hum Genet* 2002, **70**:124-141.

5. Gillespie KM, Nolsoe R, Betin VM, Kristiansen OP, Bingley PJ, Mandrup-Poulsen T, Gale EA: **Is puberty an accelerator of type 1 diabetes in IL6-174CC females?** *Diabetes* 2005, **54**:1245-1248.
